# n-ZnO nanorods/p^+^-Si (111) heterojunction light emitting diodes

**DOI:** 10.1186/1556-276X-7-664

**Published:** 2012-12-06

**Authors:** Jenn Kai Tsai, Jun Hong Shih, Tian Chiuan Wu, Teen Hang Meen

**Affiliations:** 1 Department of Electronic Engineering, National Formosa University, Yunlin, 632, Taiwan

**Keywords:** ZnO, Nanorods, Hydrothernal method, Heterojunction, LED, Seed layer free

## Abstract

In this study, we report the effects of thermal annealing in nitrogen ambient on the optical and electrical properties of zinc oxide (ZnO) nanorod (NR) arrays for the application in light emission diodes (LED). The single-crystalline ZnO NR array was synthesized on p^+^-Si (111) substrate without seed layer using simple, low-cost, and low-temperature hydrothermal method. The substrate surface was functionalized by hydrofluoric acid and self-assembled monolayer of octadecyltrimethoxysilane ((CH_3_ (CH_2_)_17_Si(OCH_3_)_3_). ZnO NRs were characterized by field emission scanning electron microscopy (FESEM), X-ray diffraction (XRD), and micro-photoluminescence (micro-PL). The results of FESEM and XRD indicate that single crystalline ZnO NRs with (002) preferred orientation along the substrate surface is successfully grown on functionalized p^+^-Si (111) substrate. The current–voltage and electroluminescence (EL) characteristics of the LED show that the most suitable annealing temperature ranges from 400°C to 600°C. Both PL and EL spectra show broadband emissions, ultraviolet and visible (green-yellow) light. The white-like light emission is able to be observed by naked eyes.

## Background

Zinc oxide (ZnO) is a II-VI compound semiconductor that exhibits n-type conduction due to oxygen deficiency or zinc excess. It has a direct wide bandgap of 3.37 eV, large exciton binding energy of 60 meV at room temperature, which is much higher than that of GaN of 25 meV, and defect emissions that cover the whole visible range [1]. Among them, one-dimensional ZnO nanostructures have attracted considerable attention for the possible integration with existing technology and easy controllability compared to other nanostructures, by virtue of their advantages in photoelectric properties. In contrast to ZnO and GaN, ZnO has large single crystals that can be grown easily. It has a very high melting point of 1,977°C, making it difficult to achieve melt to growth. Therefore, the excitons in ZnO are more thermally stable at room temperature [2]. The toxicity and environmental impact are very low than most of the other semiconductors; ZnO is actually used as a UV-blocker in sun lotions or as an additive to human and animal food [3]. In this regard, ZnO structures such as nanowires [4], nanorods, and nanoflower [5] have been synthesized using different methods under different conditions. Thus, ZnO is a promising material for optoelectronic applications as light emitting diodes [6], laser diodes [7], field-effect transistor [8], solar cells [9], light detector [10], and so on. In the present study, the simple, low-cost, and low-temperature hydrothermal method was used to synthesize ZnO nanorod (NR) arrays on the p^+^-type silicon substrate without seed layer. After annealing in nitrogen, ZnO emits both green and yellow emissions. In recent years, Leung et al. [11] reported that annealing at temperature as low as 200°C can result in significant enhancement of the UV emission, while the yellow defect emission can be almost entirely suppressed by annealing in reducing atmosphere at 600°C. Recently, Lee et al. [12] have reported the preparation of ZnO nanorod/p^+^-GaN heterojunction light emission diode (LED) by hydrothermal method. In this paper, we investigated on improving the optical properties of the ZnO NRs by annealing the samples in nitrogen ambient under different temperatures. Thermal annealing of ZnO NRs under different temperatures also shows changes in the emission wavelength.

## Methods

### Experimental details

The single crystalline ZnO NR arrays were synthesized on p^+^-Si (111) substrate without seed layer using the simple, low-cost, and low-temperature hydrothermal method. The p^+^-Si (111) substrate was employed due to its lattice constant (3.84 Å) which is close to that of ZnO (001) (3.25 Å). Also, it possesses a hexagonal lattice structure. In this study, the substrate resistivity is below 0.01 to 0.05 Ω cm, and the doped density is about 10^18^ cm^−3^. The substrate surface was light etching by hydrofluoric (HF) acid for 10 min at room temperature after standard cleaned process to prepare -H terminals on the substrate. Water contact angle increased from about 41° to 63° measured by a contact angle meter showing that the substrate surface was covered by -H terminals. Subsequently, it was then rinsed with deionized (DI) water and dried in N_2_ gas flow. The Si substrate was placed together into a teflon pressure cooker with a cup of 0.2 ml octadecyltrimethoxysilane (ODS) and sealed with a cap. The cooker was placed in an oven at 150°C for 1 h to form ODS self-assembled monolayer (SAM) on the Si substrate surface. Water contact angle of ODS SAM surface increased to 81°, indicating that almost the entire substrate was covered by ODS. ZnO NRs were grown on the SAM/p^+^-Si substrate surface in a mixture solution consisting of DI water, 0.08 M zinc nitrate hexahydrate (Zn(NO_3_)_2_·6H_2_O), and 0.08 M hexamethylenetetramine (C_6_H_12_N_4_, (HMT)) for 1 h at 95°C. Subsequently, each sample was thoroughly rinsed with DI water to remove any residual chemicals and salts, and dried at 45°C for many hours in the cooker. The crystalline grains were formed on the SAM, firstly. Zn^2+^ was continuously supplied from Zn(NO_3_)_2_·6H_2_O solution. There is almost no gradient of concentration mean that the concentration of zinc precursor is uniform from the roots to the top of rods, which results in the formation of ZnO nanorods with flat tops. After the reaction is finished, the highly oriented ZnO rods have been successfully prepared on seed-free Si substrate. Finally, n-ZnO NRs/p^+^-Si heterojunction was obtained. ZnO NRs grown on Si substrates were annealed in nitrogen gas with 1 × 10^2^ Torr for 20 min at various temperatures (200°C to 800°C) in a rapid thermal annealing furnace. The photoresist (PR) was used as an insulating layer to fill up the space between the ZnO NRs by spin coating. The extra PR was remove by the oxygen plasma treatment in plasma striper; the etch power is 450 W acting for 40 min. ITO film was sputtered onto the top of ZnO as anticathode. The working pressure was 1.5 × 10^−2^ Torr with a sputtering power of 30 W and the sputtering time was 1 h. The thickness of ITO layers was about 30 nm.

The morphologies and structural properties of ZnO NRs were examined by field emission scanning electron microscopy (FESEM) and X-ray diffraction (XRD), respectively. The optical properties and crystal defects were determined by room-temperature (RT) micrometer photoluminescence (micro-PL) using a He-Cd laser (*λ* = 325 nm) as the excitation source. The electroluminescence (EL) spectra were obtained by monochromator equipped in micro-PL system and using a current source (Keithley 2400, Keithley Instruments, Inc., OH, USA). The current–voltage (I-V) characteristics of the devices were measured by a source meter (Keithley 2400, Keithley Instruments, Inc., OH, USA).

## Materials

The materials used in this research are as follows:

A. ODS (molecular formula, C_21_H_46_O_3_Si; appearance, colorless liquid; molar mass, 374.67 g/mol; density, 0.883 g/cm^3^; melting point, 16°C to 17°C) was purchased from ACROS (Thermo Fisher Scientific Inc., MA, USA).

B. Zinc nitrate hexahydrate (molecular formula, Zn(NO_3_)_2_·6H_2_O; appearance, colorless and deliquescent crystals; molar mass, 297.49 g/mol; density, 2.065 g/cm^3^; melting point, 36.4°C) was purchased from Hayashi Pure Chemical (Osaka, Japan).

C. HMT (molecular formula, (CH_2_)_6_ N_4_; appearance, white powder; molar mass, 140.186 g/mol; density, 1.33 g/cm^3^; boiling point, 280°C) was also purchased from Hayashi Pure Chemical (Osaka, Japan).

## Results and discussion

**Figure 1:**
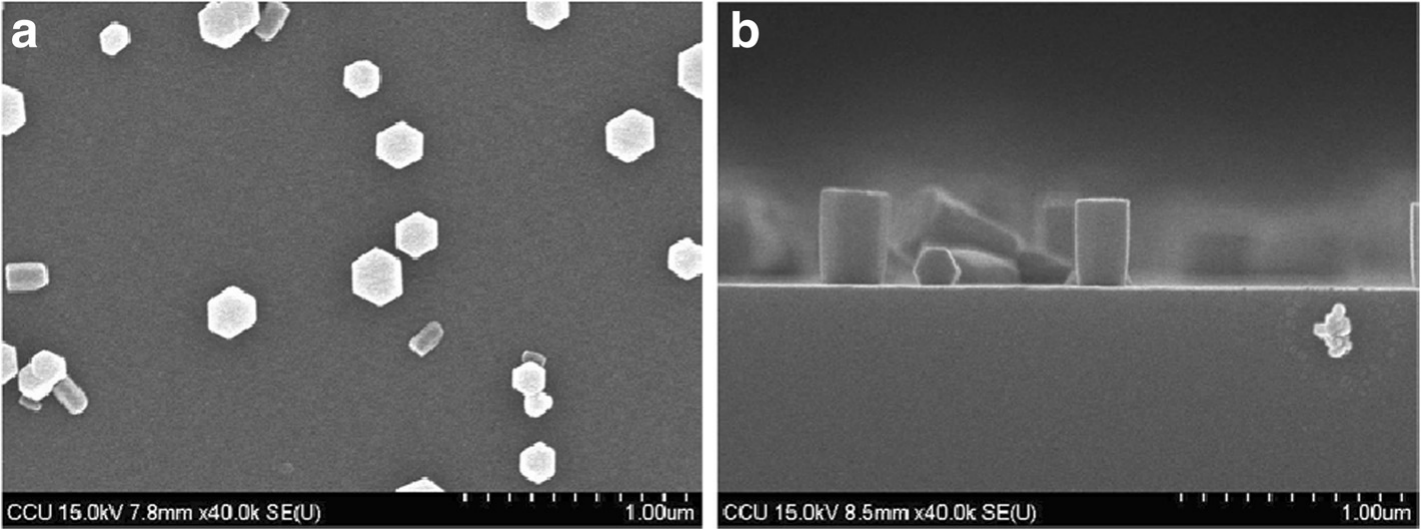
**The SEM images of as-grown ZnO NRs on p^+^-Si (111) substrate.** The diameter and length is about 223 and 392 nm, respectively.

Figure 1 shows the SEM top view and cross-section images of as-grown ZnO NR arrays. The highly oriented ZnO NR array was obtained successfully on the seed-free layer of p^+^-Si (111) substrate. The planar view reveals that ZnO NRs grow with a hexagonal shape. NRs diameter and length was about 223 and 392 nm, respectively. The ZnO NRs only occupying 4% surface area indicates that the density of ZnO NRs is very low. We have carried out the XRD analysis of these samples. In order to reduce the transmission impedance of ZnO NRs, the growth time was reduced to only 1 h for getting short ZnO NRs; it is maybe another reason of sparse NRs.

**Figure 2:**
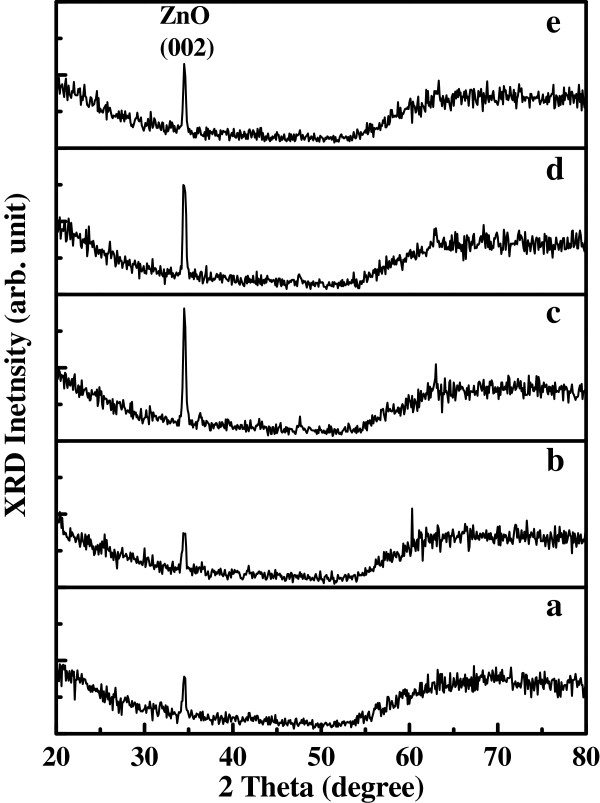
**XRD patterns of the ZnO nanorod arrays grown on *p***^**+**^**-Si(111)substrate.** (**a**) as-grown and after thermal annealing process at various temperatures (**b**) 200°C, (**c**) 400°C, (**d**) 600°C, and (**e**) 800°C, respectively.

Figure 2 shows XRD patterns of ZnO NRs grown on p^+^-Si (111) substrate with SAM. XRD intensity was weak because the NRs were small and short. After annealed in nitrogen gas at different temperatures, the samples still have major peak indexed as (002) of the wurtzite structure of ZnO. The result indicates that the NRs are preferentially oriented in the *c*-axis direction.

**Figure 3:**
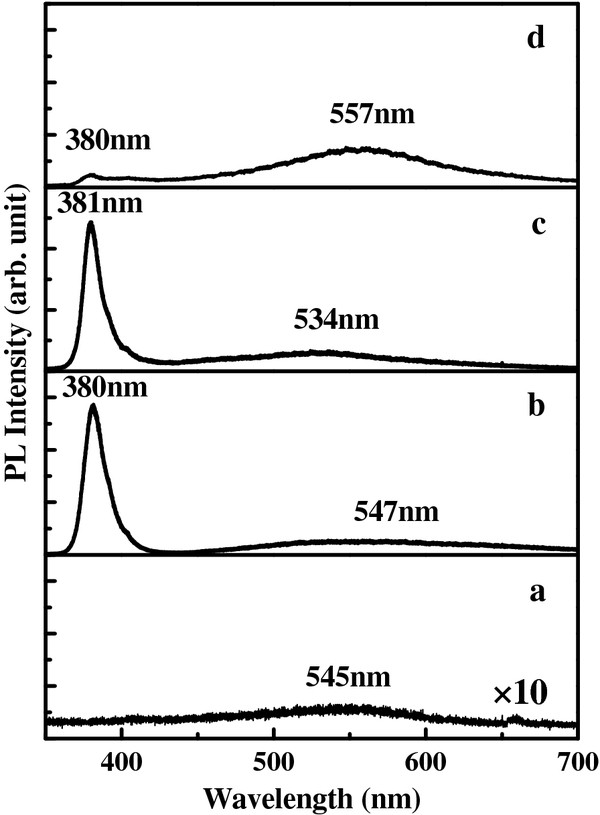
**Micro**-**PL spectra of annealed ZnO NRs grown on the p**^**+**^**-Si(111)substrates.** (**a**) 200°C, (**b**) 400°C, (**c**) 600°C, and (**d**) 800°C, respectively.

In the micro-PL system, the spot diameter of laser-beam is less than 1 μm. Therefore, the measured range covered only a small amount of ZnO NRs. Figure 3 (a) to (d) shows the micro-PL spectra of various samples which were annealed at 200°C, 400°C, 600°C, and 800°C, respectively. It shows that ZnO NRs have an ultraviolet emission and a broad band defect emission. Curve (a) showing only very weak green-yellow emission (peak at 545 nm) was detected because the number of ZnO NRs was sparse compared to SEM image and XRD results. It is maybe from the only defect emission. Curve (b) shows the ultraviolet emission at 380 nm and green-yellow emission at 547 nm. The UV emission corresponds to the near-band-edge emission, and the visible emission (green to yellow) is usually attributed to deep level defects such as oxygen vacancies and zinc interstitials, though the actual origins of visible emission still remain controversial, especially for green emission. Curve (c) also shows the ultraviolet emission at 381 nm and green-yellow emission at 534 nm. Curve (d) shows the weak ultraviolet emission at 380 nm and strong yellow emission at 557 nm. We believe that the green-yellow emission was from the defect of the interstitial Zn (Zn_i_) and anti-site O (O_Zn_) [13]. The green-yellow emission was enhanced as the annealing temperature increases in the nitrogen gas ambient.

**Figure 4:**
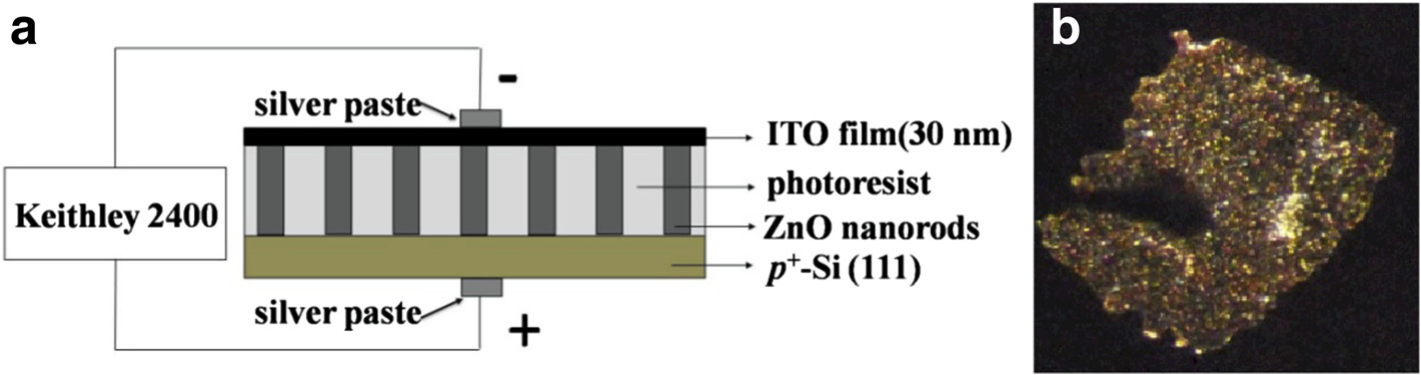
**Heterojunction LED structure and light emission of LED.** (**a**) The schematic diagram of the heterojunction LED structure and (**b**) the photography of the light emission of LED under forward bias at the dark room. n-ZnO NRs in this heterojunction light emitting diode was annealed in nitrogen ambient at 200°C. Light emissions of all the samples in this study were observed by naked eyes in the dark room.

Figure 4a shows the schematic diagram of these heterojunction LED structure, and Figure 4b shows the photography of n-ZnO NRs/p^+^-Si (111). ZnO NR array was treated by annealing at 200°C, under injected forward current. The stably white-like light emission was clearly observed in the dark room at room temperature by the human naked eye. Obviously, it emits spot light at overall surface. The dark area at the left part near the center in the photograph was the electrode made by silver paste and gold wire.

**Figure 5:**
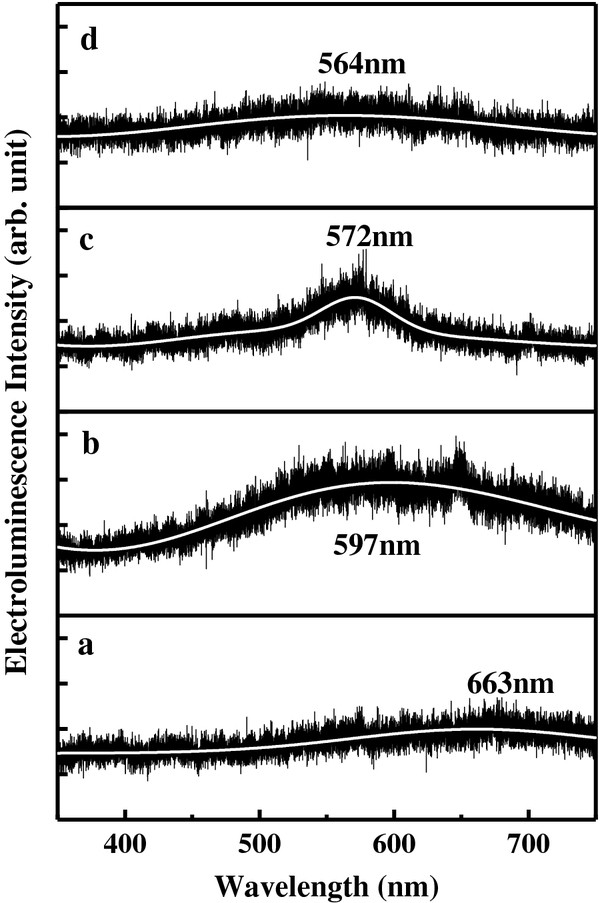
**EL spectra of n-ZnO NRs/p**^**+**^**-Si(111)heterojunction LEDs.** (**a**) 200°C, (**b**) 400°C, (**c**) 600°C, and (**d**) 800°C, respectively.

Figure 5 shows the EL spectrum of n-ZnO/p^+^-Si (111) LED. Four major emission wave bands of EL spectrum could be observed, (a) red emission (633 nm), (b) yellow-orange (597 nm), (c) green-yellow (572 nm), and (d) green-yellow (564 nm) which belong to the sample annealed 200°C, 400°C, 600°C, and 800°C, respectively. Due to low density of NRs, the emission intensity is weak. From curves (a) to (d) in Figure 5, the emission peak of ZnO NR arrays exhibits the blue shift and reduces the width of peak as the annealing temperature increases. It may indicate that the amount of defects in the ZnO NR was fewer by annealing treatment at nitrogen ambient.

**Figure 6:**
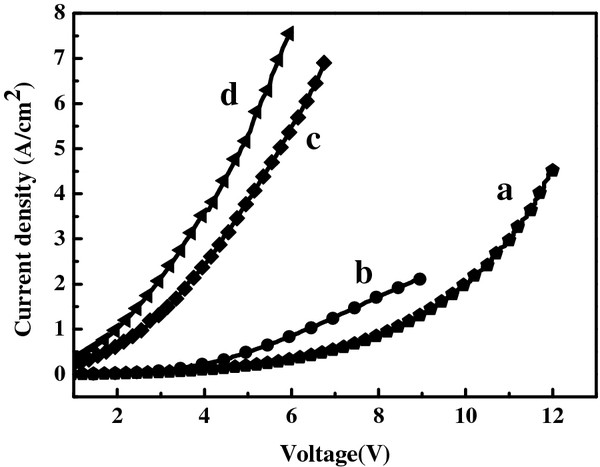
**I-V characteristics of n-ZnO NRs/p**^**+**^**-Si heterojunction.** (**a**) 200°C, (**b**) 400°C, (**c**) 600°C, and (**d**) 800°C. The turn-on voltage were 9.8, 3.7, 3.2, and 3.1 V, respectively.

A current source meter, Keithley 2400, was employed to measure the I-V characteristics of the n-ZnO nanorod/p^+^-Si (111) heterostructured LEDs shown in Figure 6. In here, we compared the electrical characteristics with each heterostructured LED by current density. Ye et al. [14] reported that the ZnO/Si heterojunction exhibits a type II band alignment, determined by the Anderson model. Since the large difference between the conduction band offset and the valence band offset, the energetic barrier was much lower for electrons than holes. Thus, only electrons are able to get across the depletion layer below turn-on voltage. This electron injection behavior subsequently leads to the wider depletion layer in the n-ZnO side, and the forward current becomes space-charge limited causing early current saturation. This behavior disappears as the voltage is above turn-on voltage. In this regime, hole injection commences and dominates the current across the junction due to higher concentration of holes in the heavy-doped Si side than that of electrons in ZnO. Therefore, the turn-on voltages were 9.8, 3.7, 3.2, and 3.1 V for the samples annealed at 200°C, 400°C, 600°C, and 800°C, respectively. This result indicates that the annealing treatment could improve the interface between ZnO NRs and p^+^-Si substrate surface due to the decreasing turn-on voltage while annealing temperature is increasing. The minimum turn-on voltage in this study is close to the result in the report of Ye et al., and it could have injected more current density under the same bias into the LED structure after being annealed at higher temperature that could improve the crystallization of ZnO which is common with the results of PL and EL.

## Conclusions

In the study, we have successfully synthesized n-ZnO NR arrays on p^+^-Si (111) substrates without seed layer by hydrothermal method growth technique for the application in heterojunction LEDs. SEM images show that the ZnO NRs are hexagonal crystallographic pillars standing vertically with respect to the substrate surface. XRD spectra results also indicate that the ZnO NRs have the wurtzite structure and grown vertically on the Si substrates along (002) direction. From the results of PL, EL, and I-V measurement, the crystallization was improved after annealing in N_2_ ambient at a temperature around 400°C to 600°C. The stable white-like light emission was observed by the naked eyes in the dark room at room temperature. For the future work, we will increase the density of NRs to enhance the emission intensity of n-ZnO NRs/p^+^-Si LED.

## Abbreviations

DI: Deionized; EL: Electroluminescence; FESEM: Field emission scanning electron microscopy; HF: Hydrofluoric; HMT: Hexamethylenetetramine; I-V: Current–voltage; LED: Light emission diode; NR: Nanorod; ODS: Octadecyltrimethoxysilane; PL: Photoluminescence; PR: Photoresist; SAM: Self-assembled monolayer; XRD: X-ray diffraction; ZnO: Zinc oxide.

## Competing interests

The authors declare that they have no competing interests.

## Author’s contributions

JKT designed this work and wrote this manuscript. JHS carried out the preparation of the samples, XRD, micro-PL, EL, and I-V measurements. TCW and THM helped in carrying out the FESEM measurements. All authors read and approved the final manuscript.

## Author’s information

JKT obtained his Ph.D. in Physics in 2004 from the National Sun Yet-Sen University (NSYSU). He has been working at the Department of Physics, National Sun Yat-Sen University as a post doc and at the Department of Electronics Engineering and Computer Science, Tung-Fang Institute of Technology as an assistant professor. Currently, he is an assistant professor with the Department of Electronic Engineering, National Formosa University, Yunlin, Taiwan. His research interest includes the synthesis of wide band gap material, GaN base, ZnO, and TiO_2_ and their application in photoelectron, light emitting diode, sensor, and solar cell. JHS obtained his masters degree in 2012 from the Department of Electronic Engineering, National Formosa University, Yunlin, Taiwan. Now, he is waiting for wok. TCW received his Ph.D. from the Department of Electrophysics, National Chiao Tung University (NCTU), Hsinchu, Taiwan in 2007. Currently, he is an assistant professor in the Department of Electronic Engineering, National Formosa University, Yunlin, Taiwan. His current research interests include semiconductor physics, superconducting thin films, and nanotechnology. THM was born in Tainan, Taiwan on August 1, 1967. He received his BS degree from the Department of Electrical Engineering, National Cheng Kung University (NCKU), Tainan, Taiwan in 1989, and his MSc and Ph.D. degrees from the Institute of Electrical Engineering, National Sun Yat-Sen University (), Kaohsiung, Taiwan in 1991 and 1994, respectively. Currently, he is a professor in the Department of Electronic Engineering, National Formosa University, Yunlin, Taiwan. His current research interests include semiconductor physics, optoelectronic devices, and nanotechnology.
